# Reduction of permanent pacemaker implantation by using the cusp overlap technique in transcatheter aortic valve replacement: a meta-analysis

**DOI:** 10.1007/s00392-022-02150-8

**Published:** 2023-01-19

**Authors:** Elias Rawish, Sascha Macherey, Dominik Jurczyk, Toni Pätz, John Jose, Thomas Stiermaier, Ingo Eitel, Christian Frerker, Tobias Schmidt

**Affiliations:** 1 University Hospital Schleswig-Holstein, Medical Clinic II, University Heart Center Lübeck, Lübeck, Germany; 2 DZHK (German Centre for Cardiovascular Research), Partner Site Hamburg/Kiel/Lübeck, Lübeck, Germany; 3 University of Cologne, Faculty of Medicine and University Hospital Cologne, Clinic III for Internal Medicine, Cologne, Germany; 4 Cardiac Valve and Structural Heart Disease Clinic, Christian Medical College Hospital, Vellore, India

**Keywords:** TAVR, Transcatheter aortic valve replacement, Pacemaker, Conduction disturbance, Cusp overlap projection

## Abstract

**Background:**

The need for permanent pacemaker (PPM) implantation is a common complication after transcatheter aortic valve replacement (TAVR). Deep implantation position is a risk factor for PPM implantation. Thus, in the field of self-expandable (SE) transcatheter heart valves (THV) cusp overlap projection (COP) technique was implemented to reduce parallax, allowing a more precise guidance of implantation depth.

**Aims:**

This meta-analysis aims to report the outcome of patients undergoing TAVR with SE THV using COP versus conventional implantation technique (CIT).

**Methods:**

Systematical search in MEDLINE and EMBASE yielded five observational controlled studies comparing both implantation techniques for the SE Evolut prosthesis (Medtronic Intern. Ltd., CA, USA) and fulfilling the inclusion criteria for meta-analysis.

**Results:**

Totally, 1227 patients were included, comprising 641 who underwent COP and 586 CIT TAVR. Incidence of post-procedural need for PPM implantation was significantly lower in COP group (9.8% vs 20.6%; OR = 0.43; *p < *0.00001). This was accompanied by significantly higher implantation position in COP group (mean difference distance from distal end of the intraventricular portion of the THV to the non-coronary cusp (NCC): − 1.03 mm; *p = *0.00001). Incidence of new-onset left bundle branch block did not differ. Regarding procedural and 30-day mortality, technical success, post-procedural aortic regurgitation, and rates of multiple device implantation, no difference between COP and CIT was found.

**Conclusion:**

COP is an effective and safe implantation technique to reduce the need for a permanent pacemaker implantation during TAVR with SE Evolut prosthesis.

**Graphical abstract:**

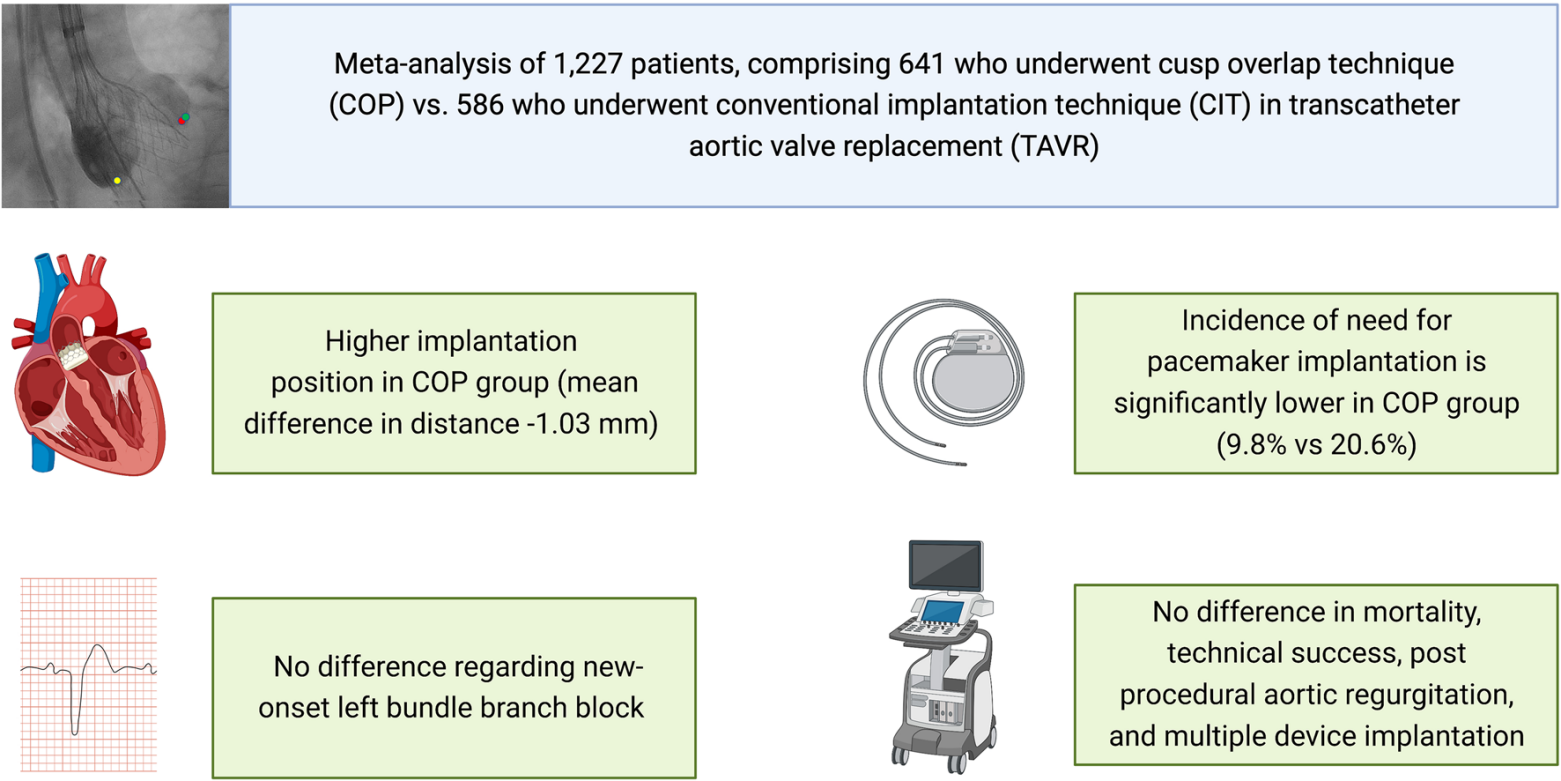

**Supplementary Information:**

The online version contains supplementary material available at 10.1007/s00392-022-02150-8.

## Introduction

**Fig. 1:**
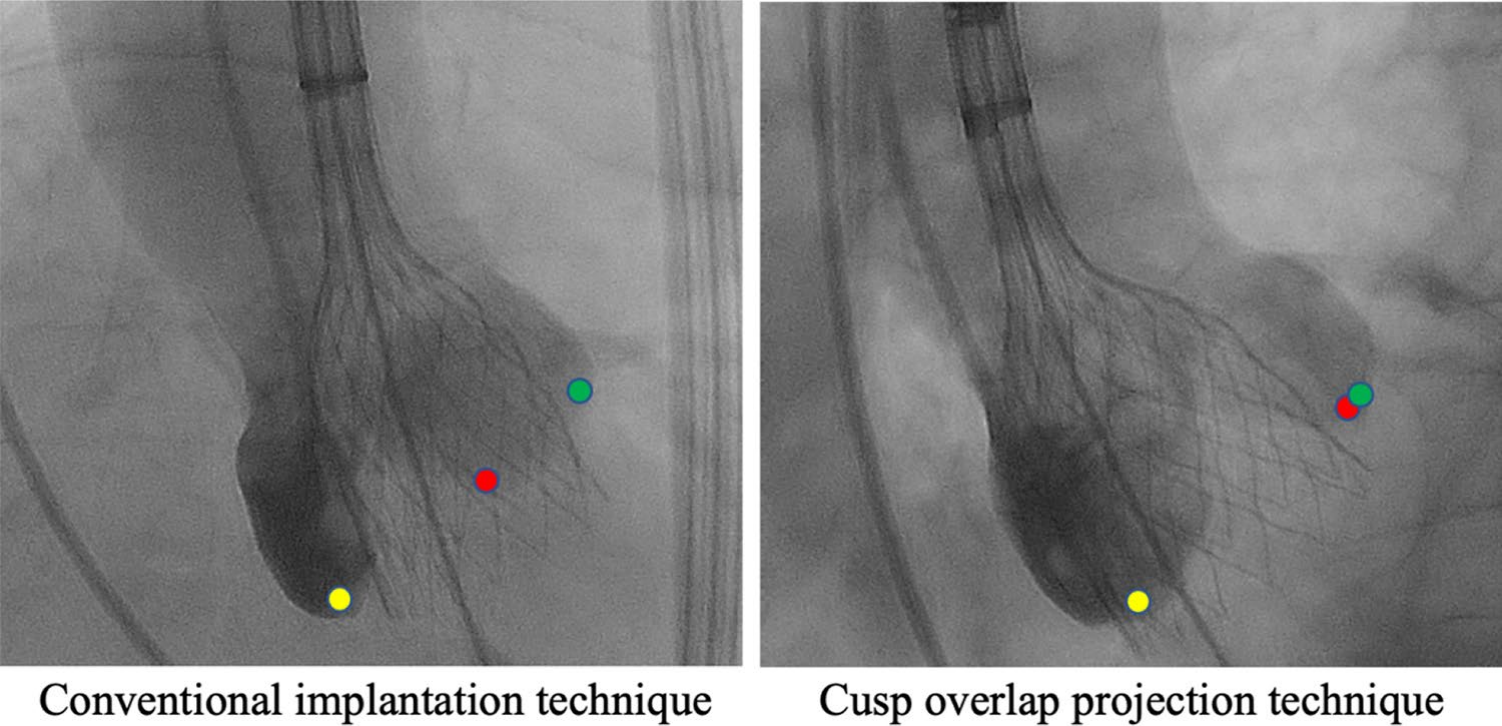
Classical three-cusp coplanar projection is shown on the left side. Cusp overlap projection technique is shown on the right side in fluoroscopy. The green dot marks the left coronary cusp, the red dot marks the right coronary cusp, and the yellow dot marks the noncoronary cusp

Aortic stenosis constitutes the most common primary valve disease requiring treatment in industry nations [1]. Since the first transcatheter aortic valve replacement (TAVR) in 2002 [2], the number of TAVR procedures has significantly expanded. To date, TAVR constitutes the first line therapy option for patients suffering severe aortic stenosis being at high or intermediate risk for surgery or being older than 75 years of age [1]. Although a recent registry analysis of 106,749 patients demonstrated a low 30-day mortality of 2.2% [3], supporting the safety of the procedure, several complications remain to be considered. Importantly, analyses revealed a permanent pacemaker (PPM) implantation prevalence of up to 22% following TAVR, as atrioventricular (AV) conduction abnormalities may occur due to the proximity of the AV conduction system to the aortic root [4, 5]. Considering the underlying mechanism for the occurrence of a high-degree AV block after TAVR, tissue damage predominantly occurs during the positioning and expansion of the prosthetic valve [6]. Transcatheter heart valve (THV)-induced traumatic lesions to the atrioventricular membrane or interventricular septum tissue may cause hematoma, edema, and necrosis, leading to conduction abnormalities [7]. Several factors have been identified to increase the risk for high-degree AV block following TAVR such as previous conduction disturbances, heavily calcified valves, increased age, and self-expanding (SE) valves [8, 9]. However, regardless of prosthesis type, the depth of implantation constitutes one of the most often identified procedural factors determining need for PPM implantation, with deeper implantation being associated with a higher risk of new conduction disturbances [10–12]. Thus, implantation of THVs at a high position can reduce the risk of PPM implantation by minimizing contact of the valve frame with the base of the membranous septum. This must be weighed against the risk of THV embolization to the ascending aorta following a higher implantation. THVs are usually positioned in a coplanar fluoroscopic projection with using three cusps view during the procedure (conventional implantation technique, CIT). Though CIT cannot fully ensure high implantation position using SE valves like the Evolut prosthesis (Medtronic Intern. Ltd., CA, US), they can deploy asymmetrically from the noncoronary cusp (NCC) toward the left coronary cusp (LCC). Therefore, the cusp overlap projection technique (COP) with overlapping of the LCC and the right coronary cusp (RCC), isolating the NCC, have been devolved to unravel the left ventricular outflow track, reducing parallax, and allowing more accurate measurements. Thereby, COP enables more predictable and higher implantation of THVs, enhancing distance of THV positioning relative to the membranous septum (Fig. 1) [13, 14]. Regarding the novelty of COP, the present meta-analysis is the first aiming to assess (a) the impact of COP on PPM implantation rate and (b) safety of COP compared with CIT considering 30-day mortality and device success following TAVR with SE Evolut prosthesis.

## Methods

The present meta-analysis was realized according to a pre-specified protocol and explicit reproducible routine for literature search and synthesis according to the preferred reporting items for systematic reviews and meta-analyses (PRISMA) guidelines [15] and has been registered in the PROSPERO database. The study selection was independently conducted by two reviewers (E.R. and D.J.). In case of any disagreement, a consensus was achieved by engagement of the senior author (T.S.). All observational studies comparing PPM implantation rates after TAVR using COP or CIT have been included. Articles published in English were eligible for analysis. Publications written in other languages were excluded. We performed an electronic search of the bibliographic databases MEDLINE and EMBASE. Thereby, the following search terms were used “(< cusp > OR < cusp overlap > OR < overlap >) AND (< tavr > OR < tavi > OR < transcatheter aortic valve replacement > OR < transcatheter aortic valve implantation >)”. 30-day new PPM implantation incidence after TAVR was preliminary defined as primary outcome. Secondary end points were 30-day all-cause mortality, technical success, and implantation depth. All data were collected from text, tables, and figures. We collected the following data from the original trials: first author, year of publication, country, operation period, number of patients enrolled, study design, patients’ age, sex distribution, prosthesis type, outcome definition and implantation success rate, Society of Thoracic Surgeons (STS) score, implantation depth (ID) of THV, PPM implantation rate, new-onset of LBBB, incidence of post-procedural aortic regurgitation, periprocedural stroke rate, incidence of multiple valve implantation or valve embolization, hospital stay duration, and 30-day mortality.

### Statistical analysis

Random effects meta-analyses were performed using the Mantel–Haenszel method for dichotomous data to estimate pooled odds ratios (ORs) and 95% confidence intervals (CI). Weights were calculated by using Mantel–Haenszel methods.

Furthermore, the *I*^2^ statistic to quantify possible heterogeneity was calculated (*I*^2^ < 30%: low heterogeneity; 30% < *I*^2^ < 75%: moderate heterogeneity; *I*^2^ > 75%: considerable heterogeneity; Review Manager 5.4, Nordic Cochrane Centre, Cochrane Collaboration). We defined *p < *0.05 as a statistically significant difference. The level of evidence of the original trials was evaluated according to the criteria of the Oxford University [16]. To assess the studies’ quality, we judged the individual and overall risk of bias. Initially, we intended to use the risk of bias tool provided by the Cochrane Collaboration, but as we were only able to include nonrandomized trials, we changed to the ROBINS‐I (Risk Of Bias In Non‐randomized Studies ‐ of Interventions) tool [17]. Two reviewers independently judged the risk of bias according to the given criteria (E.R. and D.J.).

## Results

**Fig. 2:**
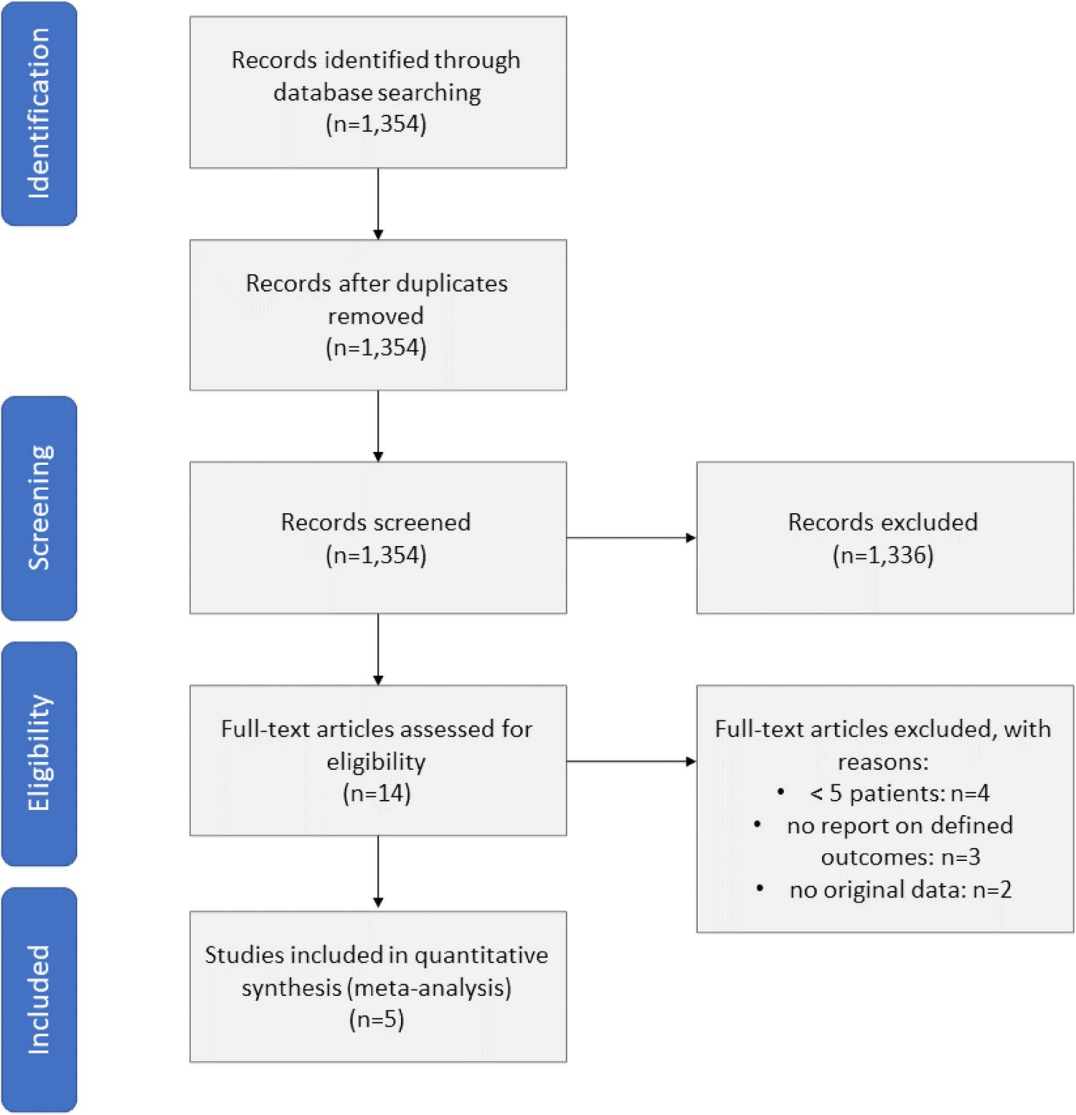
Flowchart diagram of information through the different phases of the systematic review

After removing duplicates, the search strategy led to 1354 references on September 1, 2022. In total, 1336 records were excluded after screening the abstracts as they were not thematically relevant. Following thorough revision of the 14 left references, five observational studies comparing both implantation techniques for the self-expandable THV prosthesis fulfilled the inclusion criteria for quantitative analysis (Fig. 2). There were no randomized controlled trials eligible. All references were case–control studies either with or without propensity score matching. According to the criteria of the Oxford University, these references represent a level of evidence of 4 [16].

A total of 1227 patients undergoing CIT‐TAVR (*n = *586) or COP-TAVR (*n = *641) were eligible for quantitative analysis (Table 1) [18–22]. There were no significant differences between the groups considering median age, sex, STS score, prior AV block type 1, prior LBBB, prior RBBB, calcium score of the aortic valve, LVOT calcification, and perimeter-derived annulus diameter (Table 1). A preexisting PPM was an exclusion criterion for all analyzed studies except for Maier et al. The rate of preexisting PPM was 8.0% in both groups following propensity matching [22].

**Table 1:** Baseline characteristics of analyses comparing COP‐TAVR and CIT-TAVR

References	Countries	Operation period	Number of patients	Prosthesis	Study design	Median age, y	Sex: male patients, %	STS score, %	Prior AV block type 1, *n* (%)	Prior LBBB, *n* (%)	Prior RBBB, *n* (%)	Calcium score of the aortic valve (AU), mean ± SD	LVOT calcification % of the perimeter of the LVOT	Perimeter-derived annulus diameter (mm), mean ± SD
Mendiz et al. [18]	Argentina, Costa Rica, Slovenia	08/2019–06/2020	257 COP: 156 CIT: 101	Medtronic Evolut R and Evolut PRO	Multicentric retrospective case series	COP: 79.6 CIT: 79.8	COP: 50.6% CIT: 48.5%	COP: 5.9 CIT: 5.8	COP: 3 (1.9) CIT: 1 (0.9)	COP: 15 (9.6) CIT: 10 (9.9)	COP: 18 (11.5) CIT: 10 (9.9)	COP: 3298.6 ± 916.5 CIT: 3231.2 ± 1040.3	COP: 5.18 CIT: 5.94	–
Pascual et al. [19]	Spain	03/2017–11/2020	226 COP: 113 CIT: 113	Medtronic Evolut R and Evolut PRO	Single-center, observational and prospective study	COP: 83.1 CIT: 83.8	COP: 61.1% CIT: 59.3%	COP: 5.24 CIT: 4.91	COP: 13 (11.5) CIT: 22 (19.47)	COP: 16 (14.16) CIT: 18 (15.93)	COP: 16 (14.16) CIT: 17 (15.04)	COP: 2.993 ± 99 CIT: 3.224 ± 1.438	–	COP: 24.15 ± 2.83 CIT: 24.08 ± 2.46
Doldi et al. [21]	Germany	04/2019–10/2021	122 COP: 61 CIT: 61	Medtronic Evolut	Single‐center, retrospective case series	COP: 82.6 CIT: 83.3	COP: 75.4% CIT: 83.6%	COP: 3.5 CIT: 3.1	COP: 4 (6.6) CIT: 7 (11.7)	COP: 9 (14.8) CIT: 8 (13.3)	COP: 11 (18.0) CIT: 4 (6.7) *p = *0.11	COP: 2.905 ± 1.551 CIT: 2.507 ± 1.444	COP: 11.3 CIT: 1.8 *p = *0.11	–
Pascual et al. [20]	Spain	02/2015–02/2021	322 COP: 161 CIT: 161	Medtronic Evolut	Bicentric, prospective propensity‐matched analysis	COP: 81.8 CIT: 82.5	COP: 49.1% CIT: 43.5%	COP: 4.3 CIT: 4.2	COP: 20 (12.4) CIT: 21 (13.0)	COP: 22 (13.7) CIT: 22 (13.7)	COP: 22 (13.7) CIT: 25 (15.5)	COP: 2,614.7 ± 1,475.2 CIT: 2,666.9 ± 1,307.6	–	COP: 23.7 ± 0.2 CIT: 23.7 ± 0.17
Maier et al. [22]	Germany	01/2016–04/2021	300 COP: 150 CIT: 150	Medtronic Evolut	Single-center, prospective propensity‐matched analysis	COP: 81.3 CIT: 81.3	COP: 58% CIT:58%	COP: 4.3 CIT: 4.8	–	COP: 9 (6.0) CIT: 10 (6.7)	COP: 8 (5.3) CIT: 7 (4.7)	–	COP: 53.3 CIT: 53.3	COP: 24.5 ± 2.2 CIT: 24.7 ± 2.5

– indicates data not reported; *AV* atrioventricular; *CIT* conventional implantation technique; *COP* cusp overlap projection technique; *LBBB* left bundle branch block; *LVOT* left-ventricular outflow tract; *RBBB* right bundle branch block; *STS* Society of Thoracic Surgeons; *TAVR* transcatheter aortic valve replacement

**Fig. 3:**
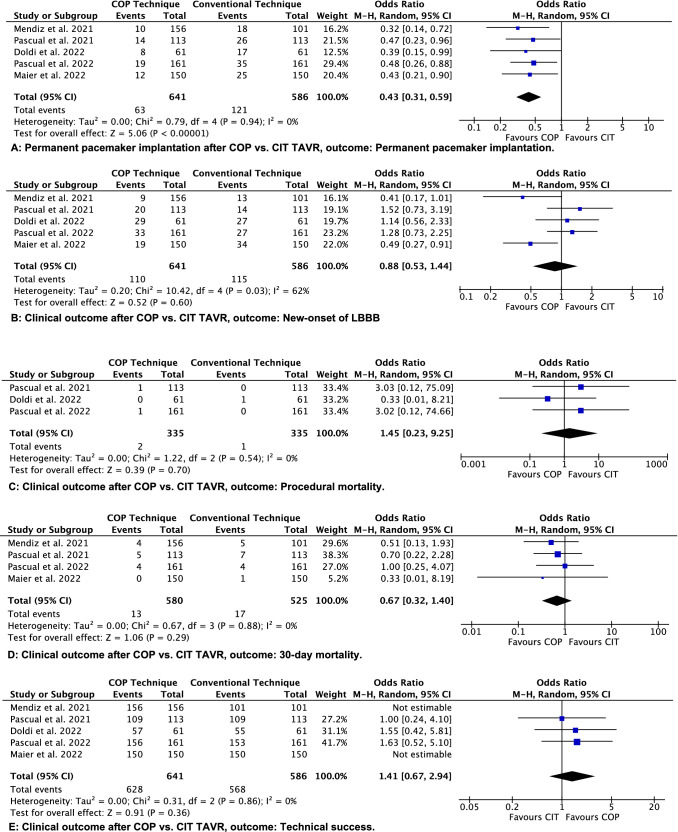

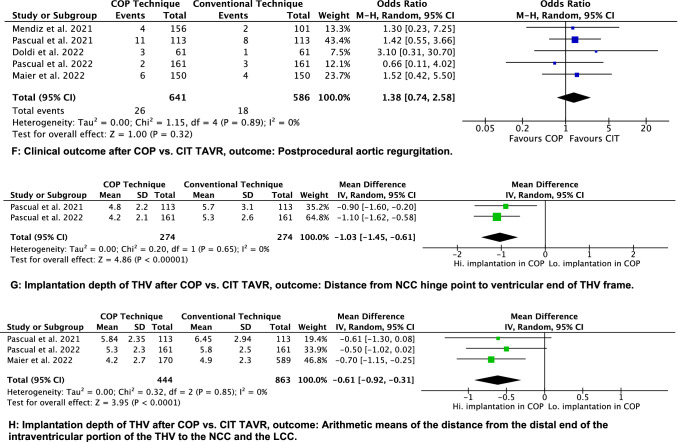
Comparison of conventional implantation technique (CIT) and cusp overlap projection technique (COP) transcatheter aortic valve replacement (TAVR). M–H indicates Mantel–Haenszel

A total of 121 of 586 participants treated with CIT‐TAVR and 63 of 641 patients undergoing COP‐TAVR underwent a post-procedural new PPM implantation (*N = *5 trials; 9.8% vs 20.6%; OR = 0.43; 95% CI [0.31, 0.59]; *p < *0.00001; *χ*^2^ = 0.79; *I*^2^ = 0%) (Fig. 3A, Table 2). However, with respect to the incidence of new-onset left bundle branch block no difference was found (*N = *5 trials, 17.2% vs 22.7%, OR = 0.88; 95% CI [0.53, 1.44]; *p = *0.60; *χ*^2^ = 10.42; *I*^2^ = 62%) (Fig. 3B). Regarding procedural mortality no significant difference between the groups was seen (*N = *3 trials, OR = 1.45; 95% CI [0.23, 9.25]; *p = *0.70; *χ*^2^ = 1.22; *I*^2^ = 0%) (Fig. 3C). The all cause 30‐day mortality was 3.2% for patients undergoing CIT‐TAVR and 2.2% for patients undergoing COP-TAVR. This difference was not significantly different between both groups (*N = *4 trials; OR = 0.67; 95% CI [0.32, 1.40]; *p = *0.29; *χ*^2^ = 0.67; *I*^2^ = 0%) (Fig. 3D).

**Table 2:** Procedural outcome of reports comparing COP‐TAVR and CIT-TAVR

References	Implantation success definition and rate	Periprocedural PPM implantation, *n* (%)	New-onset LBBB, *n* (%)	30-day mortality, *n* (%)	Any stroke after 30-days, *n* (%)	Aortic regurgitation, *n* (%)	Multiple device implantation, *n* (%)	Conversion to open heart surgery, *n* (%)	Valve embolization, *n* (%)	Hospital stay, days	ID NCC mean, mm	ID Mean of LCC/NCC mean, mm
Mendiz et al. [18]	Device success: COP: 97.4% CIT: 95.1% Technical Success: COP: 100% CIT: 100%	COP: 10 (6.4) CIT: 18 (17.8)	COP: 9 (5.8) CIT: 13 (12.9)	COP: 4 (2.6) CIT: 5 (4.9)	COP: 1 (0.6) CIT: 0	Moderate: COP: 4 (2.5) CIT: 2 (2) Severe: COP: 0 CIT: 0	–	–	COP: 1 (0.64) CIT: 0 (0)	Mean: COP: 2.7 CIT: 2.9	COP: 3.49 CIT: –	COP: 4.57 CIT: –
Pascual et al. [19]	Technical success: COP: 96.5% CIT: 96.5%	COP: 14 (12.4) CIT: 26 (23.0)	COP: 20 (20.62) CIT: 14 (14.74)	COP: 5 (4.42) CIT: 7 (6.19) Procedural mortality: COP: 1 (0.88) CIT: 0	COP: 11 (9.73) CIT: 8 (7.08)	Moderate: COP: 4 CIT: 2 Severe: COP: 7 (6.19) CIT: 6 (5.31)	COP: 4 (3.54) CIT: 4 (3.54)	–	COP: 0 CIT: 1 (0.88)	Mean: COP: 7.51 CIT: 7.06	COP: 4.77 CIT: 5.71	COP: 5.84 CIT: 6.45
Doldi et al. [21]	Technical success: COP: 93.4% CIT: 90.2%	COP: 8 (13.1) CIT: 17 (27.9)	COP: 29 (47.5) CIT: 27 (44.3)	COP: – CIT: – Procedural mortality: COP: 0 CIT: 1 (1.6)	COP: - CIT: - Procedural stroke: COP: 2 (3.3) CIT: 3 (4.9)	> I°: COP: 3 (4.9) CIT: 1 (1.6)	COP: 0 (0) CIT: 1 (1.6)	COP: 0 (0) CIT: 1 (1.6)	–	–	–	–
Pascual et al. [20]	Technical success: COP: 96.9% CIT: 95.0%	COP: 19 (11.8) CIT: 35 (21.7)	COP: 33 (20.5) CIT: 27 (16.8)	COP: 4 (2.5) CIT: 4 (2.5) Procedural mortality: COP: 1 (0.6) CIT: 0	COP: - CIT: – Procedural stroke: COP: 2 (3.3) CIT: 3 (4.9)	Severe: COP: 6 (3.7) CIT: 7 (4.4)	COP: 5 (3.1) CIT: 5 (3.1)	–	COP: 1 (0.6) CIT: 3 (1.9)	Median: COP: 4 CIT: 6	COP: 4.2 CIT: 5.14	COP: 5.3 CIT: 5.8
Maier et al. [22]	Technical success: COP: 100% CIT: 100%	COP: 12 (8.0) CIT: 25 (16.8)	COP: 19 (12.8) CIT: 34 (22.9)	COP: 0 (0) CIT: 1 (0.7)	COP: 7 (4.7) CIT: 3 (2.0)	> I°: COP: 6 (4.0) CIT: 4 (2.7)	COP: 2 (1.3) CIT: 0 (0.0)	–	COP: 0 (0) CIT: 0 (0)	Mean: COP: 8.4 CIT: 10.3	–	COP: 4.2 CIT: 4.9

– indicates data not reported; *CIT* conventional implantation technique; *COP* cusp overlap projection technique; *LBBB* left bundle branch block; *PPM IMPLANTATION* permanent pacemaker; *TAVR* transcatheter aortic valve replacement; *VARC* VALVE Academic Research Consortium

Technical success was achieved in 96.9% of participants treated with CIT‐TAVR and 97.97% of patients undergoing COP‐TAVR (*N = *5 trials, OR = 1.41; 95% CI [0.67, 2.94]; *p = *0.36; *χ*^2^ = 0.31; *I*^2^ = 0%) (Fig. 3E). There was no difference in the incidence of moderate and severe post-procedural aortic regurgitation (*N = *5 trials, 3.1% vs 4.1%, OR = 1.38; 95% CI [0.74, 2.58]; *p = *0.32; *χ*^2^ = 1.15; *I*^2^ = 0%) (Fig. 3F).

There was no significant difference found in rates of multiple device implantation (*N = *4 trials, 2.1% vs 2.3%, OR = 1.05; 95% CI [0.44, 2.50]; *p = *0.91; *χ*^2^ = 1.55; I^2^ = 0%) (Suppl. Fig. 1B) and THV embolization (*N = *4 trials, 0.76% vs 0.34%, OR = 0.51; 95% CI [0.10, 2.57]; *p = *0.42; *χ*^2^ = 0.89; *I*^2^ = 0%) (Suppl. Fig. 1B). The need for conversion to open heart surgery was only reported by one study (1 case in CIT vs. no cases in COP group) [21].

Regarding the distance from NCC hinge point to ventricular end of THV frame, a significantly higher implantation was achieved in COP group compared to CIT (*N = *2 trials, 4.41 mm vs 5.44 mm; mean difference − 1.03 mm, 95% CI [− 1.45, − 0.61]; *p = *0.00001; *χ*^2^ = 0.20; *I*^2^ = 0%). Arithmetic means of the distance from the distal end of the intraventricular portion of the THV to the NCC and the LCC were significantly different, pointing to a higher THV implantation in COP group (*N = *3 trials, 4.89 mm vs 5.51 mm; mean difference -0.61 mm, 95% CI [− 0.92, − 0.31]; *p = *0.00001; *χ*^2^ = 0.32; *I*^2^ = 0%) (Fig. 3G, H). A trend toward a shorter hospitalization in COP group was not significantly different (*N = *3 trials, 5.55 vs 6.15 days; mean difference − 0.60 days, 95% CI [− 1.82, 0.62]; *p = *0.33; *χ*^2^ = 7.32; *I*^2^ = 73%) (Suppl. Fig. 1C).

To evaluate the individual risk of bias of each study, we used the ROBINS‐I tool, as described in the methods section. The results are summarized in Table 3. In brief, most studies included comparing COP with CIT were retrospective case series or non-randomized prospective observational studies resulting in serious to moderate risk of bias in most categories. Thoroughly, we rate the overall risk of bias for these studies as “moderate.”

**Table 3:** Risk of bias assessment according to risk of bias tool to assess non-randomized studies of interventions (ROBINS-I) tool

References	Confounding	Selection of participants	Classification of interventions	Deviations from intended interventions	Missing data	Outcome measurement	Selection of reported results
Mendiz et al. [18]	Moderate risk of bias	Moderate risk of bias	Moderate risk of bias	Low risk of bias	No sufficient information	Moderate risk of bias	No sufficient information
Pascual et al. [19]	Moderate risk of bias	Moderate risk of bias	Moderate risk of bias	Low risk of bias	No sufficient information	Moderate risk of bias	No sufficient information
Doldi et al. [21]	Moderate risk of bias	Moderate risk of bias	Moderate risk of bias	Low risk of bias	No sufficient information	Moderate risk of bias	No sufficient information
Pascual et al. [20]	Low risk of bias	Moderate risk of bias	Moderate risk of bias	Low risk of bias	No sufficient information	Moderate risk of bias	No sufficient information
Maier et al. [22]	Low risk of bias	Moderate risk of bias	Moderate risk of bias	Low risk of bias	No sufficient information	Moderate risk of bias	No sufficient information

## Discussion

### Main findings

This is the first meta-analysis regarding observational studies comparing COP and CIT.

Main findings of this meta-analysis are as follows:

- COP is associated with a significant decreased post-procedural need for PPM implantation (9.8% vs 20.6%)
- COP enables a higher implantation depth compared to CIT (mean difference -1.03 mm distance from NCC to THV)
- Incidence of new-onset LBBB was not different between the groups
- Quantitative analysis showed no significant differences in 30‐day mortality, technical success, and rate of post-procedural aortic regurgitation
- Reduction of post-procedural need for PPM implantation did not lead to a significantly shorter hospitalization in COP group

### Implantation depth and conduction disturbances

Considering that implantation depth of THV is an important risk factor for the need of PPM implantation following TAVR [23], it is consistent that the higher implantation position that can be achieved by COP (4.41 mm vs 5.44 mm from NCC to ventricular end of THV frame) leads to a lower PPM implantation rate. Regarding the ID of balloon-expandable THVs, a mean ID of 3.2 ± 1.9 mm is reported for the SAPIEN-3 THV using CIT. However, the COP is a special implantation technique for Evolut THV prosthesis. Therefore, a direct comparison to other TAVI prostheses is not sui. However, Sammour et al. described a new implantation technique for the balloon-expandable THV using a RAO/CAUD projection. In this analysis the authors reported a higher deployment with an ID of 1.5 ± 1.6 mm [24]. For SE ACURATE neo THV mean ID of 5.0 ± 1.0 mm has been reported using CIT. Also here, absolute values of ID cannot be directly compared with the SE Evolut prosthesis we analyzed, since the THVs have different frame designs, implantation positions and radial forces.

Surprisingly, the rate of new-onset LBBB is unchanged despite higher THV implantation position in the COP group of our meta-analysis. Therefore, one can assume that the higher implantation achieved by COP reduces the chance of serious damage to the atrioventricular bundle, but the anatomical proximity between THV and the left bundle branch remains so close that no overall effect with respect to LBBB can be seen, as minor damage to the conduction system of the left ventricle still occurs to a comparable extent in COP and CIT. Regarding the result of a meta-analysis including 4756 patients showing that LBBB after TAVR is associated with an increased risk of high-degree AV Block (HAVB) occurring later on, as well as an increased cardiovascular mortality [25], long-term clinical assessment after COP should be evaluated in the future. Furthermore, long-term data would be of interest as it is known that PPM implantation may lead to a negative effect on left ventricular function, which may have an impact on long-term prognosis after TAVR by using COP or CIT.

Of note, differences in implantation depth were limited and should be considered in relation to angiographic spatial resolution (roughly 0.2 mm) and parallax issues that may persist even after careful assessment with computer tomography (CT) and COP use. Moreover, the impact of implantation depth on conduction disorders according to MS length has not been fully studied. In this context, post-TAVR CT could provide a precise measurement of the achieved implantation depth and its relation to MS depth. However, these data are missing in our current meta-analysis as these were not systematically reported in the included trials.

### Procedure safety

Considering the procedural- and 30-day mortality, technical success rate and post-procedural aortic regurgitation rate, COP can be regarded as a safe implantation technique irrespective of the higher THV implantation position. Of note, COP did not yield an increase of valve embolization rate. Nevertheless, it has to be stated that a higher THV implantation position can increase the risk for unsuccessful coronary cannulation after TAVR [26]. Therefore, careful selection of patients undergoing COP to achieve an implantation position as high as possible is required, to balance the risk for unfavorable coronary reengagement after TAVR as well as the risk for HAVB and subsequent PPM implantation. In addition, a commissural alignment implantation technique could also be used for increasing the chance of coronary access after TAVR.

Interestingly, despite the increased pacemaker incidence in the CIT group, we found no significant differences in the duration of hospitalization. This may be attributed to the fact that PPM implantation constitutes a routine procedure with a low complication rate, which only slightly increases the duration of treatment [27].

### Limitations

This meta‐analysis underlies methodological and content‐related limitations. First, only a low level of evidence could be identified. As randomized controlled trials are still missing, retrospective case series or prospective observational studies represent the current evidence of comparing COP with CIT in TAVR. However, several strategies were applied to minimize the risk of bias during the meta-analysis. All published abstracts and full‐text articles were considered, but non-peer reviewed data (e.g., from conference presentations) were not included. We planned to calculate not only forest, but also funnel plots to assess publication bias. As we did not include the minimum of 10 studies in statistical analysis of any outcome, funnel plot calculation was not appropriately feasible. Nevertheless, we assess the risk of publication bias as moderate to low, overall.

Our analysis is affected by a language bias, as we only considered articles published in English. According to the nature of a meta-analysis, the original studies were designed heterogeneously, with potential differences in baseline data, different risk profiles or varying clinical outcome measures as they have been defined according to VARC 2 [18–20] but also VARC 3 criteria [21]. For instance, two studies [19, 20] used the term ‘procedural success’ which, however, is not part of the VARC 2 endpoints [28], though the meaning of the term largely corresponds to the term ‘technical success’ of the VARC 3 endpoints.

Selection bias may have been present, as obviously, the effect of time is not considered in a rapidly changing field. The more recently treated patients may have had the advantage of technological and procedural advances, accompanied by increased knowledge and skills and physicians’ awareness of implantation depth as a critical issue to minimize conduction disturbances. In the context of the included observational studies in our meta-analysis, the difference observed in the rate of PPM implantation may also partiality reflect changes in overall patient management than the adoption of the COP technique.

## Conclusion

COP is an effective and safe implantation technique to reduce the need for a new permanent pacemaker implantation during TAVR with the SE Evolut prosthesis. To produce more reliable data on our research question randomized or at least larger, controlled prospective trials are needed.

## Impact on daily practice

This analysis clearly shows that performing COP in TAVR is suitable to reduce the risk for serious conductance disturbances and subsequent need for PPM implantation while being noninferior to CIT regarding technical success, procedural mortality, and post-procedural aortic regurgitation.

## Supplementary Information

Below is the link to the electronic supplementary material.

Supplementary file1 (DOCX 3094 kb)

## Data Availability

The data that support the findings of this study are available from the corresponding author, ER, upon reasonable request.

## Abbreviations

AV: Atrioventricular
CI: Confidence interval
CIT: Conventional implantation technique
COP: Cusp overlap projection
LBBB: Left bundle branch block
LCC: Left coronary cusp
NCC: Non-coronary cusp
OR: Odds ratio
PPM: Permanent pacemaker
SE: Self-expandable
STS: Society of Thoracic Surgeons
TAVR: Transcatheter aortic valve replacement
THV: Transcatheter heart valve
